# WHEN I’M 75: COLLEGE STUDENTS’ SELF-PERCEPTIONS OF AGING IN AN INTRODUCTORY GERONTOLOGY COURSE

**DOI:** 10.1093/geroni/igz038.238

**Published:** 2019-11-08

**Authors:** Sarah J Hahn, Jennifer Kinney

**Affiliations:** 1 Mercy College, Dobbs Ferry, New York, United States; 2 Miami University, Oxford, Ohio, United States

## Abstract

This presentation examines college students’ self-perceptions of aging using written essays from the assignment “When I’m 75” that was assigned at the beginning and end of the semester in an introductory gerontology course. Despite robust literature on people’s attitudes toward aging and older adults, far less is known about attitudes toward one’s own aging, especially among college students. Interpretive Phenomenological Analysis was used to analyze the students’ perception of their aging experience in their written assignment. Three overarching superordinate themes were identified: challenges of aging, proactive steps to avoid negative consequences of aging, and housing considerations. Findings suggest that after completing an introductory gerontology course, students demonstrated an understanding of some age-related changes yet still had a stereotypical understanding of what it is like to be age 75. This suggests the need to engage students in moving beyond stereotypes and to better link older age with their own future experience.

